# Exploring behavioural factors affecting nutritional supplement use among children in Honduras

**DOI:** 10.1017/S1368980021004468

**Published:** 2022-02

**Authors:** Kevin Long, Cynthia Salter, Chester Good, Carly Caughey, Kaitlin Fischer, Mark Meyer

**Affiliations:** 1 University of Pittsburgh, School of Medicine, 3550 Terrace St, Pittsburgh, PA 15213, USA; 2 University of Pittsburgh, Graduate School of Public Health, Pittsburgh, PA, USA; 3 UPMC Health Plan, Insurance Division, Centers for Value Based Pharmacy Initiatives and High Quality Health Care, Pittsburgh, PA, USA; 4 University of Pittsburgh, Pittsburgh, PA, USA; 5 UPMC Shadyside Hospital, Pittsburgh, PA, USA

**Keywords:** Dietary supplements, Nutrition programs, Child development, Honduras, Child health, Social aspects of health, Food and nutrition

## Abstract

**Objective:**

This study explored social and behavioural factors associated with a home fortification of complementary foods program among families of undernourished children in 14 rural communities in Honduras.

**Design:**

We collected and analysed survey data from a convenience sample of 196 households participating in a nutritional program using home fortification of complementary foods in 2017. The program supplied families with a soy-based atole powder fortified with micronutrients. A research team completed a face-to-face survey exploring social and behavioural factors associated with nutritional supplement use. Anthropometric measurements for participating children were abstracted from health clinic records of previous quarterly appointments.

**Setting:**

The study took place in San Jose del Negrito, Honduras.

**Participants:**

Participants were parents or guardians of children enrolled in the nutrition program.

**Results:**

Nearly half of participant families shared the nutritional supplement with other family members besides the index child, while 10 % reported using the supplement as a meal replacement for the child. Low education level of mothers was associated with improper use of the supplement (*P* = 0·005). Poorer families were more likely to share the supplement (*P* = 0·013).

**Conclusions:**

These results highlight the challenges of programs using home fortification of complementary foods in the context of food scarcity. Findings highlight the importance of increasing rural children’s overall caloric intake, perhaps by increasing access to locally available protein sources. Results also suggest transitioning nutritional programs to family-based interventions to increase overall intended compliance to nutrition programming.

Despite recent improvements in nutrition in many regions of the world, undernutrition, which includes both stunting and anaemia, remains a key health challenge in early childhood in low- and middle-income countries. WHO reported in 2016 that 22·9 % of the world’s children under age 5 suffer from stunting (defined as low height-for-age)^(1)^. Stunting remains part of the WHO monitoring framework of Sustainable Development Goal Two to eliminate hunger worldwide^(1)^. In many regions, including Latin America, declines in stunting have been the greatest in urban areas, while stunting remains more prevalent among children in rural areas^(2)^. A 2011 meta-analysis of the literature on multiple micronutrient (MMN)-based interventions showed that MMN supplementation reduces anaemia and can improve linear growth, reducing stunting, especially in areas where micronutrient deficiencies are common^(3)^. There is substantial literature exploring the potential benefits of MMN and home-based fortification interventions^(3,4)^, but less attention has been placed on associated behavioural factors that may influence supplement use and overall health impact of these programs. Additionally, the determinants for continued undernutrition among children enrolled in supplementation programs merit further exploration.

Malnutrition is well recognised as ‘a state of nutrition in which a deficiency or excess (or imbalance) of energy, protein and other nutrients causes measurable adverse effects on tissue/body form (body shape, size and composition) and function, and clinical outcomes’^(5)^. In our study, we refer to undernutrition as a deficiency in nutrition, as measured by stunting or anaemia in children. Undernutrition is linked to as many as 45 % of deaths among children under age 5 in low- and middle-income countries^(6)^. Children with severe undernutrition can experience iodine and iron deficiencies, leading to irreversible brain damage and poorer overall development^(7)^. In addition to higher risk of childhood mortality, undernourished children also can experience permanent mental impairment, attain less schooling, face lower economic productivity and are more susceptible to chronic diseases^(1)^. Factors related to undernutrition include dietary intake, socioeconomic status, maternal health, infectious diseases and environment^(7,8)^, as well as social and behavioural factors related to food and eating practices.

Interventions to alleviate child undernutrition include supplementation with micronutrient powders, lipid-based nutrient supplements or complementary food supplements^(4,9)^. Micronutrient powder supplementation provides MMNs but lacks macronutrients and therefore calories^(3)^. Complementary food supplement, also called home-based fortification, are composed of both micronutrients and macronutrients, providing some energy^(4)^. Lipid-based nutrient supplement are a type of complementary food supplement that are fortified, lipid-based products that provide calories and can also offer protein and micronutrients^(4,10)^. The efficacy of these interventions in reducing anaemia has been demonstrated in several studies^(4,9,11–14)^, suggesting potential improvement in all aspects of health including motor development, reduction of illness burden and improved anthropometric indicators^(3)^. The literature suggests that providing more than 3 micronutrients rather than specific micronutrients such as iron alone, vitamin A and zinc or vitamin A alone may improve indicators such as stunting or anaemia^(3)^.

Honduras is a Central American country of more than 9 million people, where more than half of families live in poverty and where 1 in 4 children suffers from undernutrition^(15)^. In the rural areas where this nutrition program was implemented, local diet was limited in quantity and variety; a typical meal might consist of rice, beans and tortillas. Meats are uncommon due to cost, and the poorest families often cannot afford to buy rice, so micronutrient deficiencies are likely to be common^(16)^. In 2000, an NGO began working with rural Honduran communities in San Jose, a remote mountain village of 1600 people in the El Negrito District of Yoro Province. The NGO uses a community-oriented primary care approach as the foundation for community interactions and programming, including the formation of the Health Committee of San Jose, composed of a local doctor and citizens from the surrounding communities. El Negrito is one of the poorest and least developed areas of Yoro, and most residents depend upon subsistence farming, with non-agricultural activities accounting for only 22 % of rural income^(16,17)^. In 1996, a national nutrition survey by the Ministry of Health of Honduras reported a stunting rate of 42·5 % in rural northern Honduras^(18)^. Similarly, in 2011 the NGO program data identified pervasive undernutrition among children in rural areas, with a prevalence of stunting and anaemia ranging from 30 to 40 % in the 14 rural communities surrounding San Jose.

In response to a request from the Health Committee of San Jose, in 2012 the NGO initiated a home fortification of complementary foods program in the rural communities surrounding San Jose. The NGO chose a soy-based atole powder fortified with 21 vitamins and minerals, including calcium, iron, magnesium, zinc, phosphorous, niacin, biotin and Vitamins A, B_12_, E, C and D. The serving size for the supplement is 18·75 grams per serving and includes 4 g of protein, 12 g of carbohydrates and 1 g of fat, resulting in 73 kcal per serving. Table 1 provides detailed information about the composition of the supplement. The supplement is designed to be consumed once a day as a meal supplement mixed with water or milk and eaten as porridge or drank as an atole for children ages 6 months until their 7th birthday^(19)^. Other studies using this supplement in Guatemala and other parts of Honduras had demonstrated decreases in stunting, decreases in anaemia, reductions in frequency of illnesses and improvement in cognition for children using the product^(20–22)^. In the current setting, the NGO community staff members completed home visits every 3 months with families of children enrolled in the nutrition program to distribute the supplement and to measure children’s height, length, weight and haemoglobin levels. Each parent or legal guardian received 4 packs of the supplement per child enrolled in the study every 3 months. Between 2012 and 2017, the program enrolled 1383 children in 14 communities in the Yoro region, collecting anthropometric measurements for participating children at quarterly health appointments and maintaining records of program participation and supplement distribution. Children aged out of the program on their 7th birthday. At the time of our 2017 survey, the average age of children receiving the supplementation was 44-months-old, so children had, on average, been receiving the supplement for 3 years. There were 672 enrolled children.

**Table 1:** Detailed nutritional information of the supplement

Nutritional facts	Amount per serving	% Daily value
Calories	73 kcal	
Calories from fat	9 kcal	
Total fat	1 g	1 %
Saturated fat	0 g	0 %
Trans fat	0 g	0 %
Cholesterol	0 g	0 %
Sodium	0 g	0 %
Carbohydrates	12 g	4 %
Dietary fibres	2 g	10 %
Sugars	0 g	0 %
Protein	4 g	9 %
Calcium	200 mg	
Phosphorous	150 mg	
Magnesium	40 mg	
Zinc	9 mg	
Copper	300 mcg	
Iron	12 mg	
Iodine	90 mcg	
Selenium	17 mcg	
Manganese	0·17 mg	
Vitamin A	250 mcg	
Vitamin B_12_	0·9 mcg	
Pantothenic acid	1·8 mg	
Folic Acid	160 mcg	
Vitamin E	5 mg	
Vitamin C	40 mg	
Thiamine	0·5 mg	
Riboflavin	0·5 mg	
Pyridoxine	0·5 mg	
Vitamin D	5 mcg	
Niacin	6 mg	
Biotin	8 mcg	

In 2015, a quantitative evaluation of the home fortification of complementary foods program assessed impact among 661 rural children participants in collaboration with partnering NGO. That evaluation found that program participation was associated with increased haemoglobin levels and decreased anaemia, but participating children showed no significant improvements in weight, height or BMI^(19)^. However, that unpublished evaluation did not assess for factors beyond biometric data. With this study, we sought to explore behavioural factors related to micronutrient supplementation use in the rural Honduran communities and to assess associations between these behavioural factors and nutritional status, as well as other common predictors of poor nutrition.

## Materials and methods

A cross-sectional study was conducted among participants enrolled in a home fortification of complementary foods nutritional supplement program in San Jose, Honduras. We collected and analysed household survey data using a structured survey developed in conjunction with local health clinic staff in San Jose in 2017. Inclusion criteria for the survey study were: parents or guardians of children between ages 6 months and 7 years who were currently enrolled in the program (receiving the micronutrient supplement for home consumption) and living with a guardian who regularly took them to a quarterly appointment at the health center in San Jose or who received regular home visits for health assessments. Children were excluded from the study if they had chronic diseases such as tuberculosis, asthma or cancer, a known genetic disorder such as Down’s Syndrome, problems swallowing food or reflux or frequent vomiting, as determined by an interview with the mother. Lastly, children were excluded from the study if one or both parents had a mental or physical disability that affected their care of the child or if the parents had not lived in the household for the past week since the survey included questions about feeding practices and health status of their children over the past week.

The lead investigator, a medical student fluent in Spanish, developed the survey in collaboration with the local physician at the San Jose healthcare clinic and with 2 US-based medical mentors who have worked in the community since the inception of the fortification program in 2012. Local community outreach workers reviewed the survey for relevance and cultural appropriateness. A training session on how to implement the questionnaire was completed with the entire research team to minimise measurement bias.

During a 2-week data collection period, the structured household survey was administered to a convenience sample mothers or guardians of the 672 children actively enrolled in the home fortification of complementary foods program and receiving regular supplies of the supplement. The research team, including 3 local outreach workers, completed in-person home visits in 14 rural communities in the Yoro region around San Jose del Negrito, One team member asked survey questions and another wrote participant answers on paper surveys. Parents verbally consented to being interviewed. All program participants who were contacted at their homes during the data collection period agreed to participate. Interviews lasted from 20 to 30 min. Each child’s anthropometric measurements were taken from clinic records of their previous quarterly appointment.

### Dependent variables

For the purposes of this study, stunting is defined for all children in our study as gender-specific height-for-age levels that have a *Z*-score 2 sd below the median of the reference population as determined by the WHO^(1)^. Anaemia is defined as haemoglobin levels of less than 11·0 g/dl for children under 5 years of age and less than 11·5 g/dl for children 5 years old or older^(1)^. Specific to our exploration of behaviour related to foods in the home, we asked if the micronutrient supplement was shared with others not in the program; if the supplement was cooked in foods that were used for other members of the household, and if the supplement was used in place of a meal for the child. Dichotomous-dependent variables were created, and supplement use with no sharing and no use as a meal replacement was coded as 0 while any improper use of the supplement was coded as 1.

### Explanatory variables

We collected information about feeding behaviours as well as potential risk factors for undernutrition that are suggested by previous literature to be significant in low-income countries^(4,9,11–14)^. These include mean age of introduction to solid foods, age of introduction to liquids other than formula and breast milk, length of time of exclusive breastfeeding, crowding in the home (denoted by number of people per room), mother’s education level and family wealth (indicated by family assets and monthly income). Severity of recent illnesses was defined by a symptom score. We created a modified symptom score based on the URI symptom score by Taylor *et al*.^(23)^. Given our interest in all infectious disease aetiologies in children in the study, we defined our symptom score as the sum of the total number of positive symptoms that each child had over the past 30 days from a review of symptoms including diarrhea, rhinorrhea, vomiting, cough, urinary symptoms, throat pain and fever. We also asked how many portions of the nutritional supplement the child had received in the last week and if they received the supplement the prior day.

### Statistical analysis

We analysed the data using as dependent variables the child’s nutritional status (stunting or anaemic) and behavioural use of the micronutrient supplement (sharing and using it as a meal replacement) to explore potential predictors of nutritional status and feeding practices. A descriptive analysis of the data was completed using means and standard deviations. Normality was tested using the Shapiro–Wilk test for continuous variables, with *P*-values not statistically significant (*P* > 0·05). Two-tailed *t*-tests using the continuity correction factor were used to determine if there were significant differences between child nutritional status and behavioural use groups. Chi-square analyses were used to explore associations between the categorical variables. Levene’s test of equal variance was completed to determine if there was an equal variance between the different variables. A multiple logistic regression was used to model the dependent variable as a function of the explanatory variables, estimating the OR and 95 % CI. A *P* < 0·05 was considered statistically significant.

## Results

A total of 212 in-person household surveys were completed. One survey excluded from analysis because of incompleteness and 15 others were excluded from analysis because the children had other health conditions. Final analysis included 196 enrolled children whose parent or guardian completed the in-person household survey. More than half of participants were female (54·1 %), and the average age of enrolled children (in months) was 43·5 ± 21·5 months (8·2 months to 7·1 years). Approximately one-third (34 %) of the children were born at home, and only 2 % of the children were known to be pre-term at birth.

The average monthly income of the families in the program was 1900 ± 1600 Lempiras (equating to 80 US dollars in 2017). On average households included 2·8 ± 1·5 adults and 3·9 ± 2·0 children, and homes had 1·8 ± 1·1 total rooms. The mothers of the children were on average 31·8 ± 8·5 years old, 58 % had only primary education or less and 83 % of the mothers did not have paid work for the past year. The majority of the families (59 %) did not have electricity in their homes and did not own motorised vehicles. See Table 2 for additional demographic information.

**Table 2:** Characteristics of mothers and children in the program

Individual child characteristics	*n* 196	
	Mean	sd
Age (months)	43·5	21·5
Sex		
Male		
%	45·9 %	
Female		
%	54·1 %	
Delivered at home		
%	33·8 %	
Small size at birthday		
%	8·1 %	
Symptom score	1·4	1·5
Household dietary diversity score	4·01	1·4
Stunted (HAZ below −2 sd)		
%	32·6 %	
Underweight (WAZ below −2 sd)		
%	12·5 %	
Anaemic		
%	15·9 %	
Maternal characteristics		
Mother’s age (years)	31·7	8·5
Mother’s education (years)	4·3	2·9
Relationship status		
Single		
%	12·8 %	
Married		
%	32·7 %	
Significant other		
%	53·6 %	
Widowed		
%	1·0 %	
Household characteristics		
Number of adults in the home	2·8	1·5
Number of minors in the home	3·9	2·0
Number of rooms in the home	1·8	1·2
Average number of people per room	4·4	2·3
Average income (lempiras)	1913	1615
Have electricity		
%	26·0 %	
Have transportation		
%	19·4 %	
Dirt or palm floors		
%	48·5 %	

HAZ, height-for-age *z*-score; WAZ, weight-for-age *z*-score.

Among 196 children included for analysis, 63 children were stunted (32 %) and 31 were anaemic (16 %). Children who were anaemic were more likely to be younger (33·3 ± 21·9 months-old *v*. 47·9 ± 20·7 months-old; *P* = 0·001). Mothers’ reports of sharing the supplement with other family members and/or using the supplement as a meal replacement were not significantly associated with child nutritional status. Mothers of stunted children were more likely to report giving their child the supplement every day for the past week compared to parents of children who were not stunted (82·5 % *v*. 61·4 %; *P* = 0·046). Household economic factors indicating lower socio-economic status (including number of rooms in the house and presence of dirt floor) were significantly associated with poor child nutritional status on multivariate analysis (*P* = 0·011; *P* = 0·014) (see Table 3).

**Table 3:** Multiple logistic regression analysis and associations with selected variables with stunting and anaemia

Selected variables	Stunted *n* 63	32·6 %	Not stunted *n* 130	67·4 %	Crude OR	95 % CI	*P*-value
Sex of child							
Male	31	34·8 %	58	65·2 %	–		0·799
Female	32	30·8 %	72	69·2 %	0·916	0·464, 1·806	
Age of child (months)	45·7	22·6	45·1	22·1	0·999	0·983, 1·015	0·869
Mother’s education							
Years	3·4	2·4	4·8	3·1	0·903	0·788, 1·036	0·146
Place of delivery							
Home	27	41·5 %	38	58·5 %	–		0·348
Hospital	35	27·6 %	92	72·4 %	0·720	0·362, 1·430	
Supplement use†							
Used as directed	52	40·0 %	78	60·0 %	2·247	1·014, 4·980	0·046*
Irregular use	11	18·3 %	49	81·7 %	–		
Behavioural practices							
Shared	28	31·1 %	62	68·9 %	1·187	0·588, 2·398	0·632
Did not Share	35	34·0 %	68	66·0 %	–		
Meal	8	42·1 %	11	57·9 %	0·642	0·194, 2·129	0·469
Snack	55	31·6 %	119	68·4	–		
Household characteristics							
Wealthier Floor	20	20·4 %	78	79·6 %	0·410	0·202, 0·834	0·014*
Dirt or palm floor	43	45·3 %	52	54·7 %	–		
Rooms in home	1·7	1·2	1·8	1·1	0·979	0·720, 1·333	0·893
Symptom score	1·1	1·4	1·5	1·5	0·938	0·725, 1·214	0·626

Selected variables	Anaemic *n* 31	15·9 %	Not anaemic *n* 164	84·1 %	Crude OR	95 % CI	*P*-value
Sex of child							
Male	17	19·1 %	72	80·9 %	–		0·621
Female	14	13·2 %	92	86·8 %	0·798	0·327, 1·950	
Age of child (months)	33·3	21·9	47·9	20·7	0·961	0·938, 0·983	0·001*
Mother’s education							
Years	4·1	2·4	4·4	3·0	0·916	0·746, 1·124	0·401
Place of delivery							
Home	13	20·0 %	52	80·0 %	–		0·349
Hospital	18	14·0 %	111	86·0 %	0·654	0·269, 1·591	
Supplement use†							
Used as directed	18	13·7 %	113	86·3 %	0·384	0·149, 0·993	0·048*
Irregular use	13	21·3 %	48	78·7 %	–		
Behavioural practices							
Shared	13	14·3 %	78	85·7 %	1·892	0·738, 4·849	0·184
Did not share	18	17·3 %	86	82·7 %	–		
Meal	3	15·8 %	16	84·2 %	1·771	0·332, 9·447	0·503
Snack	28	15·9 %	148	84·1 %	–		
Household characteristics							
Wealthier floor	16	16·0 %	84	84·0 %	1·398	0·533, 3·664	0·496
Dirt or palm floor	15	15·8 %	80	84·2 %	–		
Rooms in home	1·4	0·6	1·9	1·1	0·380	0·180, 0·801	0·011*
Symptom score	1·4	1·5	1·4	1·5	0·714	0·680, 1·303	0·714

* *P* is significant at < 0·05.

† Defined as use of supplement daily as directed or not daily.

Regarding the frequency of supplement use, the majority of families (76 %) reported that they had given the supplement to their child the day prior to the interview, and 67 % reported that they gave the supplement to their child every day for the past week. Only 10 % of families reported that they fed the supplement to the child as a meal replacement but nearly one-half (46 %) of families reported that they shared the child’s supply of micronutrient supplement with other members of the family. Children whose nutritional supplement was shared with other family members were less likely to receive the supplement daily (60·7 % *v*. 75·0 %; *P* = 0·010).

Additionally, fewer years of education for mothers was associated with how the supplement was used, with an OR of 0·845 (95 % CI 0·747, 0·957) for less likelihood of sharing, for each additional year of maternal schooling (*P* = 0·008). Each additional year of maternal schooling resulted in an OR of 0·507 (95 % CI 0·358, 0·718) indicating decreased likelihood of using the supplement as a meal replacement (*P* < 0·0005) for mothers with increased education. No women with a secondary education used the supplement as a meal replacement for their child.

Poorer families, (as measured by those with fewer total rooms in the home), were more likely to share the supplement (*P* = 0·031). Children that had a higher symptom score (*P* < 0·0005) were more likely to have been given the supplement as a meal replacement. See Table 4 for additional behavioural aspects of nutrition supplementation by selected variables.

**Table 4:** Multiple logistic regression analysis and associations with selected variables with behavioural aspects of nutrition supplementation

Selected variables	Shared *n* 91	46·4 %	Did not share *n* 105	53·6 %	Crude OR	95 % CI	*P*-value
Sex of child							
Male	38	42·2 %	52	57·8 %	–		0·150
Female	53	50·0 %	53	50·0 %	0·631	0·337, 1·182	
Age of child (months)	47·9	21·1	43·6	21·7	1·006	0·991, 1·020	0·463
Mother’s education							
Years	3·7	2·4	4·9	3·2	0·845	0·747, 0·957	0·008*
Place of delivery							
Home	31	47·0 %	35	53·0 %	–		0·610
Hospital	60	46·5 %	69	53·5 %	1·187	0·615, 2·291	
Supplement use†							
Used as Directed	54	40·9 %	78	59·1 %	0·413	0·211, 0·809	0·010*
Irregular use	35	57·4 %	26	42·6 %	–		
Household characteristics							
Wealthier floor	42	41·6 %	59	58·4 %	0·836	0·430, 1·627	0·598
Dirt or palm floor	49	51·6 %	46	48·4 %	–		
Rooms in home	1·6	0·9	2·0	1·2	0·707	0·516, 0·969	0·031*
Symptom score	1·4	1·4	1·4	1·5	1·095	0·877, 1·367	0·423

Selected variables	Supplement as meal *n* 19	9·7 %	Supplement as snack *n* 177	90·3 %	Crude OR	95 % CI	*P*-value
Sex of child							
Male	10	11·1 %	80	88·9 %	–		0·868
Female	9	8·5 %	97	91·5 %	0·903	0·272, 2·995	
Age of child (months)	41·9	23·0	46·0	21·3	0·991	0·965, 1·019	0·538
Mother’s education							
Years	2·2	1·6	4·6	3·0	0·507	0·358, 0·718	<0·0005*
Place of delivery							
Home	6	9·1 %	60	90·9 %	–		0·276
Hospital	13	10·1 %	116	89·9 %	2·096	0·553, 7·940	
Supplement use†							
Used as directed	13	9·8 %	119	90·2 %	1·268	0·317, 5·071	0·737
Irregular use	5	8·2 %	56	91·8 %	–		
Household characteristics							
Wealthier floor	10	9·9 %	91	90·1 %	1·812	0·499, 6·582	0·366
Dirt or palm floor	9	9·5 %	86	90·5 %	–		
Rooms in home	1·5	0·7	1·8	1·1	0·732	0·364, 1·475	0·383
Symptom score	2·7	1·7	1·2	1·4	2·240	1·461, 3·436	<0·0005*

* *P* is significant at <0·05.

† Defined as use of supplement daily as directed or not daily.

## Discussion

Supplemental nutrition programs and those involving home fortification of complementary foods are increasing throughout the world in attempts to improve child nutrition and health outcomes. For example, a 2013 review by UNICEF and the Centers for Disease Control and Prevention (CDC) identified 63 implemented interventions in 36 countries and another 28 planned interventions in 21 countries, reaching more than 17·2 million participants, mostly young children^(9)^. However, many interventions were developed in the absence of standardised program guidelines^(9)^, which is also the case for the intervention described in this research. Many programs involve MMN fortified atoles or other foods, like the one used in this study. In 2011, the CDC and UNICEF partnered to develop guidelines for designing and implementing home-based fortification interventions^(9)^. Guidelines are needed to help prevent inconsistencies between stated intervention objectives and expected outcomes as well as in translating research into practice with sustained adherence^(9)^. The results of this study provide valuable information about potential limitations of nutritional interventions that lack standardised program guidelines as well as new insight into potential feeding behaviours, related to family poverty, maternal education and child’s nutritional status, that can affect actual family use of micronutrients as well as program success.

Micronutrient supplementation has been shown to decrease anaemia and stunting in some studies, although findings are inconsistent^(3,4,9,11–14,24–28)^. For example, a secondary data analysis of the lipid-based supplement across 5 sites in the Horn of Africa found that supplementation was associated with reductions in anaemia but not stunting among refugee children^(25)^. A randomised controlled, community-based intervention using MMN supplementation in Guatemala found that the supplement also decreased anaemia but the source of MMN content and protein in the supplements did not affect other growth or morbidity indicators including stunting^(26)^. A micronutrient powder improved anaemia but had no effects on cognitive outcomes in young children in rural China^(27)^. Finally, a clustered randomised efficacy trial in the Democratic Republic of Congo, Zambia, Guatemala and Pakistan receiving either meat or MMN-fortified cereal interventions (similar to our study) beginning at 6 months of age did not decrease the high stunting rates in the region^(28)^. Given these inconsistent findings supporting benefits of MMN supplementation in children, a study such as ours offers insight into possible behavioural factors that can influence supplement use and thus affect health outcomes.

Our findings that socioeconomic variables in Honduras, such as the number of rooms or type of floor in the home, were associated with stunting and anaemia, reinforce similar findings from other areas of the world. For example, a cross-sectional study completed in central India and a case-control study in Mexico concluded that socio-economic factors predicted stunting among children^(29,30)^. Poverty is a fundamental core cause of undernutrition, and determining protective factors is a step in addressing the effects of poverty on children’s nutritional status^(30)^.

Our study adds a notable finding that many children in the program in northern rural Honduras were not receiving the supplement as the program intended. This finding provides additional insight into the mechanisms through which extreme poverty can detract from a home fortification of complementary foods. For example, nearly half of the children in the program received fewer servings of the supplement than recommended because the supplement distributed to their family was shared with other household members. Additionally, almost one-fifth of the children in the program ate the supplement in place of a meal, which is inconsistent with the intended use of the supplement because the supplement does not contain sufficient calories to serve as a meal replacement. These findings, although in a small sample, offer insight into behavioural and social factors that can affect the success of nutritional supplementation programs. Another notable finding of this study is that, despite the minimal impact of this nutritional supplementation program, community members ascribed value to the long-term collaboration between the local Health Committee of San Jose and the NGO for potential future programs. The results of this study, combined with information from the previous health impact analysis, have served as an impetus for investigating other potential interventions, demonstrating an ongoing and successful community-based collaboration.

It is noteworthy that lower maternal education was associated with both sharing of the supplement with other family members and use of the supplement as a meal replacement. Maternal education is recognised as a key a predictor of child health, and these findings reinforce the importance of maternal education to child health as well as to the potential success of nutrition interventions and other child health interventions. Early childhood development programs are shown to be efficacious and cost-effective when targeting education^(31)^, so providing more comprehensive education to mothers about child nutrition and optimal use of the supplement could be an effective strategy to improve outcomes for future nutrition programs.

Infectious diseases in childhood also are known to contribute to poor nutrition and growth in children^(32)^, often due to enteric fecal-oral communicable diseases^(33)^. We created a modified symptom score to predict illness burden in children over the previous month. This score did not predict nutritional status in children, but a higher symptom score predicted the use of the supplement as a meal replacement. Inadequate diet in combination with recurrent infections can alter biological pathways synergistically that results in reduced growth and cognitive development^(34)^. Children in this study facing greater burdens of infectious disease also are more likely to have poor feeding practices in the home, highlighting the increased risk of poor nutrition for these children. Increasing education on nutrition and sanitation^(33)^ for parents of children with recurrent infections could be a useful intervention for children in low-income regions.

The results of this exploratory study gathering data on the home fortification of complimentary foods should be considered within the context of several limitations. First, the study uses a convenience sample of program participants who were available during the 2-week data collection period and so may not be representative of the entire participant population. Second, the study uses respondent self-report about feeding behaviours, and their responses may be subject to recall bias as well as social acceptability bias. However, as it would be nearly impossible to observe feeding behaviours longitudinally among a sizable sample of participants, the self-reported data from the participants in this study provides a useful glimpse into what happens within the home, and contributes useful insight into the potential challenges of programs using nutritional supplements in the home. The symptom score was modified for the purposes of our study, attempting to identify total illness burden in children. We did not measure severity of symptoms and have not validated it with additional studies; however, we believe it gives insight into the burden of disease among children in this particular setting. Future studies should be done to create and validate a simple symptom score to better understand associations between illness burden and potential risk and protective factors among low-resourced children.

Also, this is a cross-sectional study, so we are unable to comment on the course of children’s nutrition over time. An additional limitation to this study, and perhaps to the feeding program overall, is the difficulty in tracking child participation in the program and following up on all program participants for timely health data collection (height, weight, blood haemoglobin) because many program participants are unable to present quarterly at the health centre for measurements.

## Conclusion

The results of this study illustrate the challenges of home-based fortification or feeding programs. First, quantifying the index child’s consumption of the home-based supplement is difficult, particularly in the context of pervasive hunger in the entire family and the overall lack of adequate calorie consumption. Second, these findings highlight the association between poverty and undernutrition as well as the family-level challenges of addressing this health issue. Our study offers insight into some family-based behavioural factors that potentially affect consumption of the supplement including feeding behaviours and social dynamics of family meals. Inadequate supplement use was more likely when the mother had low education and where the household was poorer. Future nutrition interventions might explore supplementation for the family as a whole, rather than focusing on individual children, given the common tendency for families to share resources, although the resources required for this level of nutritional assistance would be great. Future interventions should consider social, behavioural and cultural barriers to supplement use within the family that will affect who eats the product and how eating the supplement might affect consumption of other foods (such as meal replacements). Additionally, future studies should focus on individuals who continue to be undernourished even when enrolled in nutritional supplementation programs. Studies such as this one, which explores what people do at home and how they use the nutritional supplement are vital to developing interventions that successfully address child undernutrition.
